# Pulmonary lymphangitic carcinomatosis in liver carcinoma: a rare case report and literature review

**DOI:** 10.1186/1477-7819-12-66

**Published:** 2014-03-27

**Authors:** Li Zhuang, Xiangyan Liu, Chen Hu, Lin Zhang, Guoping Jiang, Jian Wu, Shusen Zheng

**Affiliations:** 1 Department of Surgery, First Affiliated Hospital, Zhejiang University School of Medicine, 79 Qingchun Road, 310003 Hangzhou, Zhejiang, China

**Keywords:** Liver carcinoma, Liver transplantation, Metastasis, Pulmonary lymphangitic carcinomatosis

## Abstract

Primary liver carcinoma is the most important malignant disease. The nodular metastatic foci of liver carcinoma are usually found in the lung, adrenal gland or abdomen after resection or transplantation. Pulmonary lymphangitic carcinomatosis (PLC) accounts for approximately 6% to 8% of metastatic cancer in the lung. The occurrence of PLC is extremely rare in liver carcinoma. Herein we report the case of a patient with PLC after liver transplantation due to liver carcinoma. PLC was confirmed by clinical manifestations, imaging studies and cytologic examination of exfoliated cells in the pleural effusion.

## Background

Primary liver carcinoma is a malignancy originating from hepatocytes and/or intrahepatic biliary epithelial cells. In China, there are more than 90 million carriers of the hepatitis B virus (HBV), accounting for 40% to 45% of HBV carriers worldwide. The high prevalence of HBV in China is the underlying reason why liver carcinoma is the malignancy with the highest morbidity and mortality rates in China. Currently, resection and liver transplantation are major strategies for the treatment of liver carcinoma. For patients with hepatic cirrhosis, liver transplantation can cure both the cancer and liver cirrhosis. However, liver carcinoma may recur or metastasize after resection or liver transplantation, mainly via the hematogenous route. Although lymphatic metastasis can occur, metastasis is usually found in the hepatic hilus, upper abdomen and retroperitoneal lymph nodes [1]. Pulmonary lymphangitic carcinomatosis (PLC) is a special manifestation of metastatic cancer in the lymphatic vessels of the lung that is characterized by diffuse or focal growth. Most PLC cases originate from adenocarcinomas. PLC is rare in liver carcinoma patients. To the best of our knowledge, no studies reported to date have described PLC after liver transplantation.

## Case presentation

**Figure 1:**
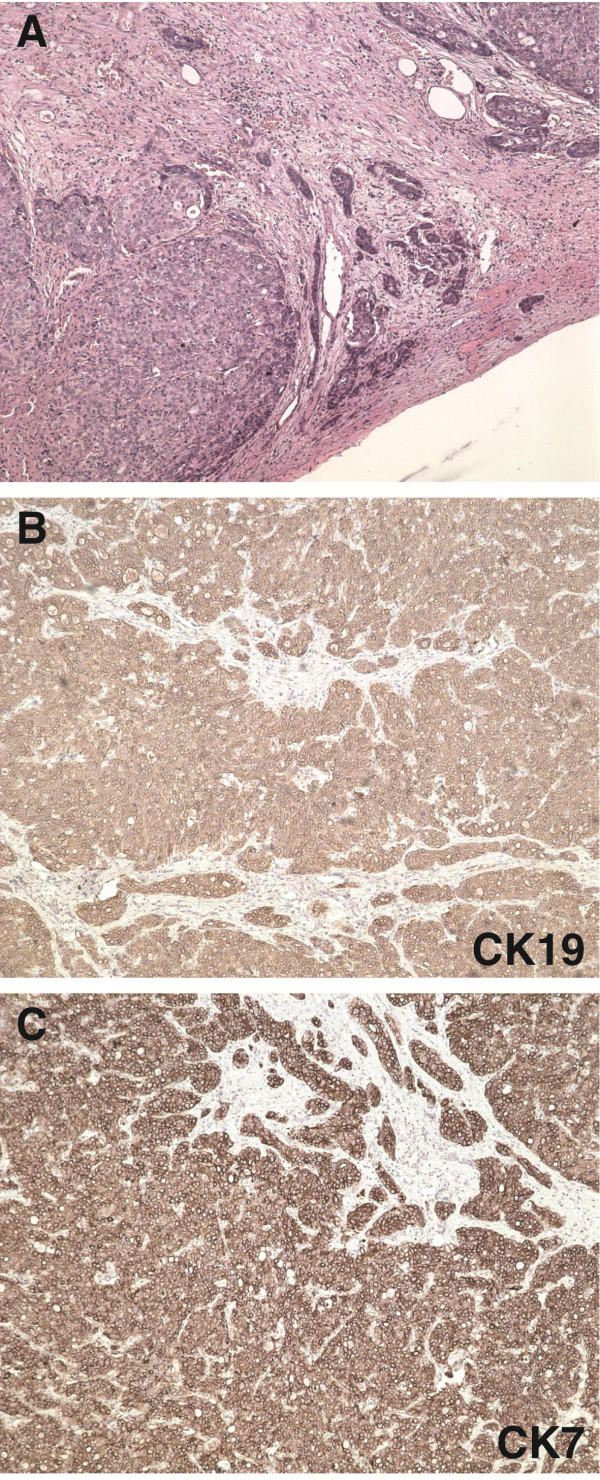
**Immunohistochemical staining of mixed liver carcinoma tissue specimens. (A)** Cancer cells were arranged in nests and showed atypia. The interstitium was rich in sinusoids, and invasive growth was noted. **(B)** Image showing cytokeratin 19 (CK19) (+). **(C)** Image showing CK7 (+). All three images are stained with hematoxylin and eosin and were scanned at 100× original magnification.

A 45-year-old man was admitted to our hospital with a complaint of repeated episodes of abdominal distension. He was diagnosed with HBV-induced hepatic cirrhosis and liver carcinoma (T3N0M0). He underwent liver transplantation without any metastasis before the operation. Pathological analysis identified a tumor (12 cm × 8 cm × 10 cm) in the right lobe of the liver, within which the cancer cells were arranged in nests and pleomorphism was seen. These findings, together with the results of immunohistochemistry, demonstrated features of mixed liver carcinoma: α-fetoprotein (+), hepatocytes (+), CD34 (+), CD19 (+), CD10 (focal, +), synaptophysin (-), chromogranin A (-) and cytokeratin (pan, +) (Figure 1). The function of the graft liver was favorable. FK506 was used alone for antirejection therapy.

**Figure 2:**
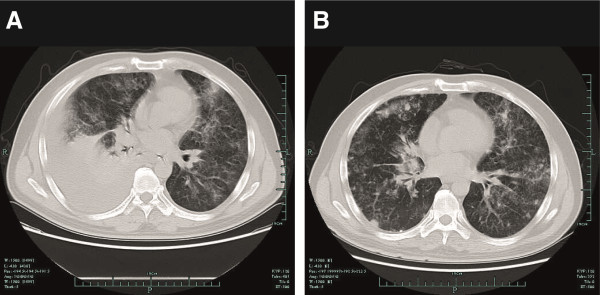
**Computed tomography scans of the lungs. (A)** Soon after the appearance of the patient’s respiratory symptoms, a computed tomography (CT) scan revealed septal thickening of the peribronchovascular interstitium, pleural effusion, segmental atelectasis in the right lower lobe of the lung and several enlarged lymph nodes in the mediastinum. **(B)** Discontinuation of anti-infection therapy, and 5 days after thoracentesis, extensive involvement of the parenchyma with septal thickening was evident, with reticulonodular densities in all lung fields.

Two months later the patient developed a dry cough of unknown etiology, and his condition deteriorated 1 week later. Expectoration was occasionally present, accompanied by chest tightness, shortness of breath and hypoxemia (75 mmHg partial pressure of oxygen). Fever and chills were absent, and the patient’s white blood cell count, neutrophil count and inflammatory factors were normal. His sputum culture was negative. Lung computed tomography (CT) suggested infectious lesions in the lung, which were characterized by interstitial changes. Right-sided pleural effusion and segmental atelectasis in the lower lobe of the right lung were noted. Several enlarged lymph nodes were identified in the mediastinum (Figure 2A). Thoracentesis was immediately performed and approximately 2,000 ml of light yellow fluid was collected. The patient’s chest tightness and shortness of breath improved significantly. Posttransplantation interstitial pneumonia was considered at first. FK506 was discontinued, and methylprednisolone (40 mg every 12 hours), caspofungin, sulfamethoxazole (SMZ) and aminophylline were administered.

**Figure 3:**
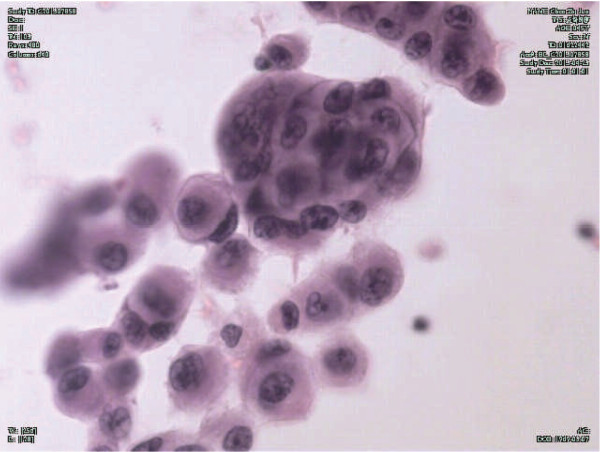
**Cancer cells among the exfoliated cells in the pleural effusion are shown.** All slides are stained with hematoxylin and eosin and were photographed under light microscope at 400 × original magnification.

Five days later a lung CT scan showed reexpansion of the right lung and diffuse exudate in the interstitium. Multiple nodules were found in both lungs (Figure 2B). Pulmonary function tests showed severe obstructive ventilatory dysfunction and moderate reduction in carbon monoxide diffusion capacity. Examination of exfoliated cells in the pleural effusion showed cancer cells (Figure 3). Positron emission tomography (PET)-CT indicated multiple nodules and patchy or cloudy shadows with high density in both lungs (maximal standardized uptake value (SUV) approximately 6.27). Several enlarged lymph nodes were found in the mediastinum, hepatic hilus and retroperitoneum (maximal SUV approximately 8.39). Moreover, lesions with increased density were found in the left third rib, the right upper femur and the left acetabulum, which were accompanied by an increase in fluorodeoxyglucose. The patient was diagnosed with PLC after liver transplantation due to liver carcinoma.

The treatment with steroid and aminophylline continued to improve the status of the patient’s interstitial lesions. Although antirejection therapy was stopped, rejection did not occur and the function of the graft liver was stable. Oral capecitabine was administered but was not effective. The patient experienced increasing chest tightness and shortness of breath, and he died as a result of respiratory failure 1 month later.

## Discussion

PLC was first described by Troisier in 1873. About 30% to 40% of malignancies may present with metastasis to the lung, and PLC accounts for approximately 6% to 8% of metastatic cancer in the lung. Most PLCs originate from adenocarcinomas, and they are most often due to lung cancer, followed by breast cancer and gastric cancer [2,3]. Patients with renal cancer, cervical cancer, thyroid cancer and melanoma rarely develop PLC [4-6]. The pathologic features of PLC include infiltration of cancer cells and interstitial edema in and around lymphatic vessels as well as infiltration of inflammatory cells caused by lymph node metastasis in the lung. The metastatic cancer in the mediastinal and pulmonary hilar lymph nodes may obstruct lymphatic drainage, resulting in retrograde migration of cancer cells into terminal lung tissues via lymphatic vessels or anterograde migration of cancer cells in the pleura into the pulmonary hilar lymph nodes through intrapulmonary lymph vessels. In addition, a cancer embolus may form in the terminal vessels of the lung due to hematogenous metastasis, which can invade the surrounding lymphatic vessels. Thus, hilar and mediastinal lymph node metastasis may be present or absent in PLC, depending on the route of metastasis of the primary cancer.

Extrahepatic metastasis of liver carcinoma is mostly found in the lung, adrenal gland, bone and central nervous system. Hematogenous spread is thought to be the most common extrahepatic metastatic route [7,8]. In the liver, lymphatic vessels run parallel to the interlobular vessels and bile duct. Lymphatic vessels can be classified as deep or superficial lymphatic collecting ducts. Superficial lymphatic collecting ducts are often found in the connective tissues of the liver capsule, and the lymph is transferred into the parasternal, paracardial and abdominal lymph nodes. The deep lymphatic collecting ducts connect to one another to form upstream and downstream trunks which transport lymph into the phrenic lymph nodes around the terminal segment of the inferior vena cava and hepatic and left gastric lymph nodes. Thus, lymphatic metastasis of liver carcinoma may be found in the hepatic hilar, upper abdominal and retroperitoneal lymph nodes because of the parallel distribution between lymphatic vessels and major abdominal blood vessels. In addition, skip metastasis may be found in several groups of lymph nodes. Overall, PLC is rare in liver carcinoma. To date, to the best of our knowledge, PLC has not been identified in patients who have undergone liver transplantation due to liver carcinoma.

PLC patients usually develop progressive dyspnea, cough, weight loss, fatigue and other symptoms, accompanied by hypoxemia, restrictive ventilatory dysfunction and diffusion dysfunction [5]. The history of cancer or surgery and characteristic features identified on lung CT scans can be used to diagnose PLC after exclusion of interstitial pneumonia, pulmonary fibrosis, sarcoidosis, pulmonary embolism, heart failure and hematogenous disseminated pulmonary tuberculosis. Biopsy and subsequent pathologic examination are not required for the diagnosis of PLC [6,9]. In the early stages, lung CT shows interstitial lesions, linear and reticular shadows and interlobar fissure thickening. Approximately one-third of PLC patients present with pleural effusion (unilateral or bilateral). Once patients develop dyspnea, other findings may be present, including irregular thickening of the tracheal vascular bundles and interlobular septa, as well as multiple beaded, small nodules of varying sizes (usually smaller than 3 mm in diameter) distributed along the interlobular septa and pleura.

In our present case report, the patient was suspected to have interstitial pneumonia due to *Pneumocystis carinii* after transplantation. Thus, management was geared toward removing the edema and treating the pulmonary infection (SMZ and caspofungin); however, the response to treatment was poor. On the basis of examination of cancer cells in the pleural effusion and the PET-CT scan, PLC was subsequently diagnosed. The positive rate of cancer cells in pleural effusion is 40% to 50%. This diagnostic accuracy rate may increase if the sediment from the pleural effusion is used for examination after being kept for 24 hours. Although biopsy via bronchoscopy, pleurocentesis, lung puncture or thoracoscopy and subsequent pathologic examination may confirm the diagnosis of PLC, biopsy increases the risk of pneumothorax. Sputum collection is relatively easy, but examination of exfoliated cells in the sputum is associated with a low positive rate [3]. Lung CT and PET-CT findings and cytology from the pleural effusion can confirm the diagnosis of PLC. Although a false-negative diagnosis of primary liver carcinoma is possible with the use of PET-CT (40% to 50%), PET-CT has favorable sensitivity in the detection of extrahepatic metastasis of liver carcinoma. Acikgoz *et al*. [10] reported that the detection rate of extrahepatic metastatic foci ≥1 cm in diameter was as high as 92.9% in liver carcinoma patients after liver transplantation. There is evidence that the specificity of PET-CT for PLC is 100% and that the sensitivity is 86%. The mean SUV in the region of PLC (1.37 ± 0.64) was significantly greater than that in the normal lung (0.5 ± 0.29) (*P* < 0.0001) [11]. Thus, combined examinations have an elevated detection rate compared to a single examination. Examinations selected according to the disease condition may significantly increase the detection rate.

To date, no effective strategies have been developed for the treatment of PLC. Currently, antitumor therapy and antispasmodic therapy of the airway with theophylline or β_2_-adrenergic receptor agonists are used. However, these treatments usually have poor efficacy, and PLC is associated with a poor prognosis. Patients usually develop progressive dyspnea and die as a result of respiratory failure and/or heart failure. Approximately 50% to 85% of PLC patients have a survival time between 3 and 6 months [12,13]. In our patient, PLC progressed rapidly because of immunosuppression after liver transplantation. Although immunosuppressive therapy was discontinued promptly, the severity of the patient’s symptoms increased rapidly and he died as a result of respiratory failure within 1 month.

In 1975, Kane *et al*. [14] reported the autopsy findings from 7,524 patients with solid cancers that originated from the prostate, breast, stomach, pancreas and liver. Involvement of the pulmonary lymphatic system by cancer cells was noted in 1,085 patients (only 1% of these patients died as a result of respiratory failure). Although PLC is rarely reported in liver carcinoma, the incidence of liver carcinoma–induced PLC might be far higher than previously reported. In addition, liver carcinoma is highly malignant and progresses rapidly. Although PLC may be present in liver carcinoma patients, these patients might die as a result of other causes, such as liver failure or hemorrhage due to cancer rupture, before the typical symptoms of PLC manifest. On the basis of our experience and previous reports, clinicians should exclude PLC when patients develop hypoxemia and interstitial pneumonia of unknown cause. PLC may cause significant deterioration of the patient’s condition. Thus, only early identification, diagnosis and treatment can prolong the survival of liver carcinoma patients with PLC.

## Conclusions

Although PLC is rare in liver carcinoma patients, cancer cells can migrate into the pulmonary lymphatic system. Early identification, diagnosis and treatment are crucial to improving the survival of PLC patients. Combined use of CT, PET-CT and pathologic examinations may significantly increase the PLC detection rate. In our patient, immunosuppressive therapy after liver transplantation caused rapid progression of PLC. Although we discontinued immunosuppressive therapy, employed strategies to improve the patient’s lung edema and administered antitumor therapy, the efficacy of the treatment was still very poor.

## Consent

Written informed consent was obtained from the patient for publication of this case report and any accompanying images. A copy of the written consent is available for review by the Editor-in-Chief of this journal.

## Abbreviations

LT: Liver transplantation; PET-CT: Positron emission tomography/computed tomography; PLC: Pulmonary lymphangitic carcinomatosis.

## Competing interests

The authors declare that they have no competing interests.

## Authors’ contributions

Li Zhuang carried out clinical data collection, participated in the sequence alignment and drafted the manuscript. XL and CH carried out pathological analysis. Lin Zhang and GJ carried out clinical data collection, JW and SZ carried out pathological analysis. All authors read and approved the final manuscript.
